# Cathepsin B trafficking in thyroid carcinoma cells

**DOI:** 10.1186/1756-6614-4-S1-S2

**Published:** 2011-08-03

**Authors:** Sofia Tedelind, Silvia Jordans, Henrike Resemann, Galia Blum, Matthew Bogyo, Dagmar Führer, Klaudia Brix

**Affiliations:** 1 School of Engineering and Science, Research Center for Molecular Life Science, Jacobs University Bremen, 28759 Bremen, Germany; 2 School of Pharmacy, Faculty of Medicine, The Hebrew University, 91120 Jerusalem, Israel; 3 Departments of Pathology and Microbiology and Immunology, Stanford University School of Medicine, Stanford, California 94305-5324, USA; 4 Universitätsklinikum Leipzig Medizinische Klinik III, 04103 Leipzig, Germany; as of June 2011: Klinik für Endokrinologie, Zentrum für Innere Medizin, Bereich Forschung und Lehre im Zentrallabor, 45147 Essen, Germany

## Abstract

**Background:**

The cysteine peptidase cathepsin B is important in thyroid physiology by being involved in prohormone processing initiated in the follicle lumen and completed in endo-lysosomal compartments. However, cathepsin B has also been localized to the extrafollicular space in thyroid cancer tissue, and is therefore suggested to promote invasiveness and metastasis in thyroid carcinomas through e.g. extracellular matrix degradation.

**Methods:**

Transport of cathepsin B in normal thyroid epithelial and carcinoma cells was investigated through immunolocalization of endogenous cathepsin B in combination with probing protease activity. Transport analyses of cathepsin B-eGFP and its active-site mutant counterpart cathepsin B-C29A-eGFP were used to test whether intrinsic sequences of a protease influence its trafficking.

**Results:**

Our approach employing activity based probes, which distinguish between active and inactive cysteine proteases, demonstrated that both eGFP-tagged normal and active-site mutated cathepsin B chimeras reached the endo-lysosomal compartments of thyroid epithelial cells, thereby ruling out alterations of sorting signals by mutagenesis of the active-site cysteine. Analysis of chimeric protein trafficking further showed that GFP-tagged cathepsin B was transported to the expected compartments, i.e. endoplasmic reticulum, Golgi apparatus and endo-lysosomes of normal and thyroid carcinoma cell lines. However, the active-site mutated cathepsin B chimera was mostly retained in the endoplasmic reticulum and Golgi of KTC-1 and HTh7 cells. Hence the latter, as the least polarized of the three carcinoma cell lines analyzed, exhibited severe transport defects in that it retained chimeras in pre-endolysosomal compartments. Furthermore, secretion of endogenous cathepsin B and of other cysteine peptidases, which occurs at the apical pole of normal thyroid epithelial cells, was most prominent and occurred in a non-directed fashion in thyroid carcinoma cells.

**Conclusions:**

Transport of endogenous and eGFP-tagged active and inactive cathepsin B in the cultured thyroid carcinoma cells reflected the distribution patterns of this protease in thyroid carcinoma tissue. Hence, our studies showed that sub-cellular localization of proteolysis is a crucial step in regulation of tissue homeostasis. We conclude that any interference with protease trafficking resulting in altered regulation of proteolytic events leads to, or is a consequence of the onset and progression of thyroid cancer.

## Background

Cathepsin B is a cysteine peptidase belonging to the papain clan C1A [1,2]. Cysteine cathepsins in general are homologous with respect to their active-site residues, i.e. cysteine (Cys) and histidine (His) forming the catalytic dyad [3]. Cathepsin B is a ubiquitously expressed member of the family of papain-like cysteine peptidases, but it is exceptional in exhibiting endo- and exopeptidase activities [4]. The main proteolytic function attributed to cathepsin B in physiology is considered in its catabolic action on proteins reaching endo-lysosomal compartments [4-6]. Hence, cathepsin B is considered to predominantly act on its substrates intracellularly, within endocytic compartments. In contrast, the extracellular occurrence of cysteine peptidases like cathepsin B is often considered pathological. Severe conditions of excessive cathepsin B-mediated degradation of extracellular matrix (ECM) components, as it is observed in osteoarthritis [7], is believed to arise when cathepsin B is secreted into the extracellular space in a non-regulated manner. Furthermore, cysteine cathepsins, and in particular cathepsin B, are considered to be involved in malignancies and cancer progression due to an increase in expression and activity in cancer cells as well as due to increased secretion from tumor-associated cells [8-12].

Because proteases display their functions by an irreversible mode of substrate cleavage, it is considered crucial to determine (i) time, (ii) location and (iii) extent of proteolytic cleavage in order to understand protease actions in physiology and pathology [1,6,13,14]. Thus, trafficking of proteases and the tight spatiotemporal regulation of proteolysis are decisive for normal or diseased functions of cells or tissues.

In the healthy thyroid gland, cathepsin B bears important functions for maintaining the differentiated state of thyroid epithelial cells in that it contributes to thyroglobulin processing and thyroxine release from the thyroid follicles [15-18]. This role of cathepsin B in thyroid physiology depends on its polarized secretion at the apical plasma membrane domain of differentiated normal thyroid epithelial cells [15,16,19]. However, cathepsin B has also been shown to be localized to the basement membrane of thyroid carcinoma cells *in situ*, where it was proposed to facilitate tumor invasiveness and metastasis through degradation of the extracellular matrix [20]. Recently, we have determined that cathepsin B is the main active cysteine cathepsin present in the human thyroid carcinoma cell lines KTC-1, HTh7 and HTh74 cells [21]. This fact, together with the suggested role of cathepsin B in malignant progression, prompted us to further analyze trafficking of cathepsin B in KTC-1 cells, a poorly differentiated papillary thyroid carcinoma cell line, and in the anaplastic HTh7 and HTh74 thyroid carcinoma cell lines. KTC-1 cells do not express thyroid stimulating hormone (TSH) receptors, thyroid peroxidase (TPO) or the sodium iodide symporter (NIS), but still express thyroglobulin [22]. Thus, these cells are characterized by both, a maintained and a lost expression of key components of the physiological thyroid hormone production machinery. In addition, despite a low expression of thyroglobulin mRNA by HTh74 cells [23], we and others have shown that this cell line still expresses functional TSH receptors [21,24].

Here, we expressed cathepsin B and its active-site mutant counterpart cathepsin B-C29A as chimeric proteins fused to the enhanced green fluorescent protein (eGFP) as visualization tag. Their transport pathways as well as their secretory release into the extracellular space of normal and thyroid carcinoma cell lines were studied with the help of activity based probes that were designed to distinguish between active and inactive cysteine peptidases within the endo-lysosomal compartments of mammalian cells [1,14,25,26]. The results of our investigations led us to conclude that protease trafficking is governed by the thyroid cell type investigated, i.e. transport resulting in polarized secretion is typical for normal, differentiated thyrocytes [1,27] whereas non-polarized transport pathways and non-directed secretion were observed in thyroid carcinoma cells. We therefore propose that cathepsin B transport to the basolateral plasma membrane domain and its secretion into the extrafollicular space as observed in follicular and papillary thyroid carcinoma tissues are features of altered trafficking routes in thyroid cancer.

## Methods

### Cell culture

Fisher rat thyroid (FRT) cells and the human thyroid carcinoma cell lines KTC-1, HTh7 and HTh74 were grown at 37°C and 5% CO_2_ in a moisturized atmosphere. KTC-1 cells were cultured in RPMI-1640 (Biowhittaker™, Verviers, Belgium), and HTh7 and HTh74 cells in Eagle´s Minimum Essential Medium (Biowhittaker™), all supplemented with 10% fetal calf serum (FCS; PerBio, Aalst, Belgium). The FRT and KTC-1 cells analyzed for the secretion of cathepsin B were grown in Coons F-12 medium (Sigma-Aldrich, Taufkirchen, Germany) containing 2.68 mg/ml sodium bicarbonate and supplemented with 5% FCS. For KTC-1 cells, a mixture consisting of 0.166 mg/ml insulin, 2 µg/ml Gly-His-Lys complex, 0.362 µg/ml hydrocortisone, 0.5 µg/ml transferrin, 1 µg/ml somatostatin and 100 µU/ml TSH (final concentrations; all from Sigma-Aldrich) was added. Barrier function and tightness of the epithelial monolayer of the thyroid carcinoma cell lines grown on permeable filter supports (pore size 0.4 mm) of Transwell inserts (Corning Costar Co., Acton, MA, USA) was estimated by measuring the trans-epithelial electrical resistance with a Millicell ERS ohmmeter (Millipore, Bedford, MA, USA). The values were corrected for background resistance measured across filters without cells.

### Indirect immunofluorescence

KTC-1, HTh7 and HTh74 cells used for indirect immunofluorescence were cultured on cover slips in 6-well plates. The cells were fixed with 4% paraformaldehyde in 200 mM HEPES, pH 7.4, for 30 minutes at room temperature followed by washing 3 times 5 minutes with 200 mM HEPES (pH 7.4) and 3 times 5 minutes with calcium- and magnesium-free PBS (CMF-PBS), i.e. 0.15 M NaCl, 2.7 mM KCl, 1.5 mM NaH_2_PO4, 8.1 mM Na_2_HPO4, pH 7.4. Permeabilization was performed with 0.2% Triton X-100 in CMF-PBS for 5 minutes at room temperature. For blocking, 3% bovine serum albumin (BSA; Carl Roth GmbH, Karlsruhe, Germany) in CMF-PBS was used for 1 hour at 37°C. The cells were incubated with an anti-cathepsin B primary antibody (Neuromics, Hiddenhausen, Germany) diluted in 0.1% BSA in CMF-PBS overnight at 4°C. After washing with 0.1% BSA in CMF-PBS, the cells were incubated with Alexa 488-conjugated secondary antibodies (Molecular Probes, Karlsruhe, Germany) for 1 hour at 37°C together with 5 µM of the nuclear counter-stain DRAQ5™ (Biostatus Limited, Shepshed, Leicestershire, UK). After washing with CMF-PBS and de-ionized water, the cover slips were mounted with embedding medium consisting of 33% glycerol, 14% Mowiol in 200 mM Tris-HCl, pH 8.5 (Hoechst AG, Frankfurt, Germany) on microscopic slides. When the thyroid carcinoma cell lines were used for F-actin labelling, they were treated as described above, but instead of antibody immunolabelling, the cells were incubated with FITC-phalloidin (3 µM, Sigma-Aldrich) for 1 hour at 37°C.

Human thyroid tissue was obtained from patients undergoing thyroid surgery and used in compliance with the Helsinki Declaration. The tissue was fixed in paraformaldehyde, embedded in paraffin and sectioned as described [28]. The tissue sections mounted on microscopic slides were de-paraffinated by washing with xylol 4 times 5 minutes followed by 5 minute-washes with decreasing concentrations of ethanol (100% to 30%) and final incubation with freshly prepared sodium borohydride (1%; Carl Roth GmbH) to reduce auto-fluorescence, and de-ionized water for 5 minutes each. Haematoxylin and eosin (0.1%; Sigma-Aldrich) (H&E) staining was performed in order to examine tissue architecture. The protocol for indirect immunofluorescence was performed as described for the cell lines above with the following modifications. Blocking with 3% BSA was performed at 4°C overnight, the permeabilization step was omitted, and the tissue sections were incubated with the secondary fluorophore-conjugated antibody for 2 hours. In addition, DRAQ5™ was used at a concentration of 20 µM. Three tissue samples from each pathological condition, i.e. from papillary and follicular thyroid carcinomas, were prepared as described above and subjected to analysis.

The immunofluorescence samples were viewed with a confocal laser scanning microscope (LSM 510 Meta; Carl Zeiss Jena GmbH, Jena, Germany) and analyzed with the LSM 510 software, Release 3.2 (Carl Zeiss Jena GmbH).

### Protein precipitation from conditioned media

Conditioned medium was collected from KTC-1 cells and proteins were precipitated with ice-cold trichloroacetic acid (TCA, 10%). The samples were incubated on ice for 30 minutes followed by centrifugation at 10 000 *g* for 10 minutes at 4^o^C. The supernatant was removed and centrifugation was repeated at the same speed and temperature for another 10 minutes. The remaining supernatant was removed and the pellet was dried in speed vacuum for 20 minutes and re-suspended in sample buffer consisting of 10 mM Tris-HCl (pH 7.6), 0.5% SDS, 25 mM DTT, 10% glycerol and 25 µg/ml bromophenol blue. The sample pH was adjusted using 1.5 M Tris-HCl at pH 8.8 (Carl Roth GmbH) before loading onto SDS-gels.

### Labelling of active cysteine cathepsins with activity based probes

HTh74 cells cultured in 6-well plates on cover slips were washed with pre-warmed PBS, i.e. 0.9% NaCl, 20 mM NaH_2_PO_4_, pH 6.8, followed by incubation with Yellow-DCG-04 (1 µM) in serum-free growth medium for 30 minutes under standard culture conditions. Washing with PBS 3 times for 5 minutes was followed by a chase period of 1 hour with complete cell culture medium and another set of washes as described above. DRAQ5™ (5 µM) was used as nuclear counter-stain and was added to the medium for the last 10 minutes of the chase period. For live-cell imaging, the cover slips with cells were transferred onto metal slide devices filled with pre-warmed medium supplemented with 20 mM HEPES to maintain neutral pH conditions during microscopy, and analyzed through confocal laser scanning microscopy as described earlier.

Active cysteine cathepsins in FRT cells were visualized using a quenched activity based probe (GB117) [25]. Transfected cells were seeded on cover slips in 6-well plates and cultured until they reached 80-90% confluence. The cells were washed once with pre-warmed PBS followed by addition of DMEM without Phenol Red (Cambrex Bio Science, Wiesbaden, Germany) supplemented with 1 μM GB117 and culturing for 3 hours under normal conditions. The live-cell imaging was performed as described above.

### SDS-PAGE and immunoblotting

Whole cell lysates of FRT cells transfected with pEGFP-N1 plasmid were obtained as follows: the cells were washed with ice cold PBS, detached with a cell scraper and collected through centrifugation for 10 minutes at 900 *g* and 4^o^C. The cells were resuspended in lysis buffer consisting of 20 mM Na_2_HPO_4_, 50 mM NaCl, 0.2% Triton X-100, pH 7.4, and supplemented with a protease-inhibitor-mix, i.e. 0.1 mM E64, 0.01 mM Pepstatin, 2 ng/ml Aprotinin, 0.02 M EDTA, followed by incubation for 30 minutes at 4^o^C on a end-over-end rotor. The supernatants were cleared through centrifugation for 15 minutes at 15 000 *g* and 4^o^C.

The Bradford assay [29] was used in order to determine the protein concentration of the samples. The proteins and a cathepsin B standard from bovine spleen (Sigma-Aldrich) were separated through SDS-PAGE on 12.5% polyacrylamide gels along with a PageRuler pre-stained protein ladder (Fermentas, St Leon-Rot, Germany) or a See Blue pre-stained standard (Novex, Frankfurt/Main, Germany), and transferred to a nitrocellulose membrane by semi-dry blotting. Unspecific binding sites were blocked with 5% non-fat milk in PBS containing 68 mM NaCl, 63.2 mM Na_2_HPO_4_, 11.7 mM NaH_2_PO_4_, pH 7.2, supplemented with 0.3% Tween (PBS-T) overnight at 4°C. Incubation with goat anti-mouse cathepsin B (Neuromics), rabbit anti-rat cathepsin B (Upstate Biotechnology, Lake Placid, NY, USA), rabbit anti-human cathepsin L (RD Laboratorien GmbH, Diessen, Germany) or rabbit anti-human β-tubulin (Abcam, Cambridge, UK) primary antibodies diluted in PBS-T was for 2 hours at room temperature followed by incubation with horseradish peroxidase-conjugated secondary antibodies (Southern Biotech, Birmingham, Al, USA) for 1 hour at room temperature. Incubation with the peroxidase substrate (ThermoScientific, Bonn, Germany) was followed by visualization through enhanced chemi-luminescence on XPosure films (ThermoScientific).

### Construction of the active site mutant cathepsin B-C29A-eGFP

**Figure 1:**
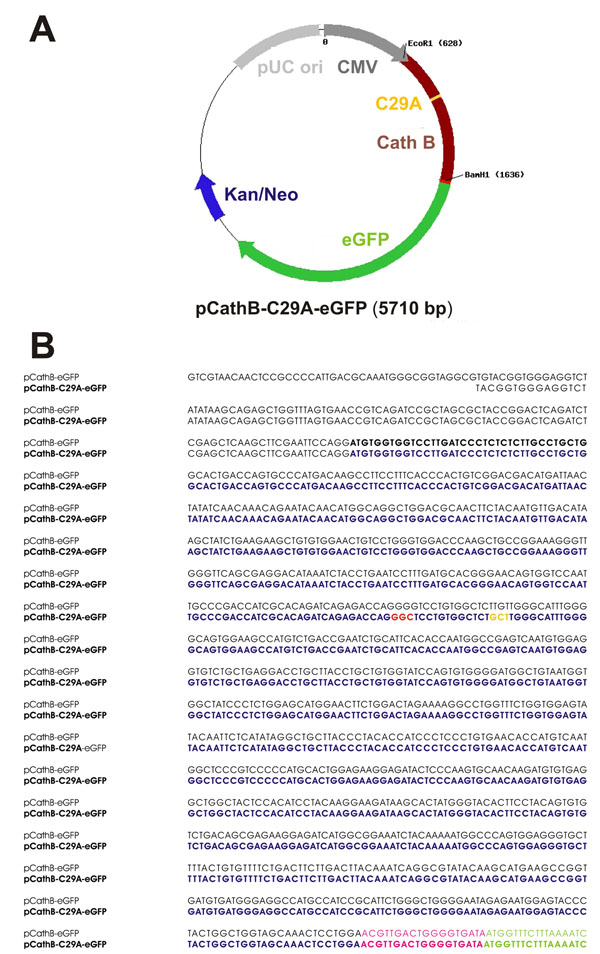
**Schematic depiction and sequence of pCathB-C29A-eGFP coding for inactive cathepsin B-eGFP chimeras.** Schematic representation (A) and nucleotide sequence (B) of the mammalian expression vector pCathB-C29A-eGFP that codes for eGFP-tagged cathepsin B containing a cysteine-to-alanine substitution in its active site (yellow) which was introduced by site-directed mutagenesis of the cathepsin B coding sequence inserted into pEGFP-N1 by using EcoR1 and BamH1 restriction sites. The plasmid bears a CMV promotor (dark grey) as well as an origin of replication (pUC ori, light gray). The eGFP coding sequence (green) is located down-stream of the cathepsin B encoding DNA minus the nucleotides encoding the C-terminal pro-peptide extension of the enzyme. In the resulting chimeric protein, cathepsin B will be fused to eGFP by a 6-amino-acids linker peptide (light red). The sequences of pCathB-eGFP [27] and the active-site mutant pCathB-C29A-eGFP were aligned by means of ClustalW (version 1.82) multi alignment tool. The sequences are identical, except for the point mutations inserted through site-directed mutagenesis, and resulting in a GGG to GGC ‘silent’ mutation (red), as well as a TGT to GCT codon exchange (orange), causing a cysteine to alanine exchange within the active-site of cathepsin B.

Cathepsin B cDNA was derived from FRTL-5 cells, a Fisher rat thyroid cell line, as described previously [27]. For site directed mutagenesis and generation of pCathB-C29A-eGFP, the CathB 49-1068 forward primer [27] was combined with a newly designed primer CB-Cys29Ala reverse, 5’ – AGA GCC ACA GGA GCC CTG GT – 3’, giving rise to the amplification of the first 300 base pairs (bps) of the cathepsin B cDNA. The CB-Cys29Ala reverse primer was constructed such that a codon was changed from TGT to GCT, causing the exchange of cysteine to alanine at amino-acid position 29 of rat cathepsin B. Next, a second PCR reaction was performed using CB-Cys29Ala forward, 5’ – GGC TCC TGT GGC TCT GCT TGG GCA TTT G – 3’, in combination with the CathB 49-1068 reverse primer [27], giving rise to the last approximately 700 bps from the 3’-end of the *Ctsb* gene. Thereby, two mutations were caused, namely the already mentioned exchange from TGT to GCT (see above) and a ‘silent’ mutation (GGG to GGC), which would not alter the amino acid composition upon translation, but resulted in the omission of a restriction site for EcoO109I. Thus, the silent mutation allowed for better analysis of cloning success. In addition, the sequences of the primers were designed such that an EcoRI as well as a BamHI cleavage site were inserted at the 5’- and the 3’-ends of the cDNA, respectively, to allow for the insertion of the complete coding sequence of cathepsin B into the pEGFP-N1 vector after fusion PCR was performed as follows and as previously described [27]. The PCR products were separated on 1% agarose gels and the DNA was stained with ethidiumbromide. The 300-bps- and 700-bps-*Ctsb* fragments were cut out, purified from the gel and used as templates for a fusion PCR reaction. Here, the CathB 49-1068 primer pair [27] was used in order to amplify full-length cathepsin B cDNA bearing the above mentioned sequence mutations. This step was repeated and gained the cathepsin B-C29A-fragment with the correct size of 1019 bps, which was excised from the gel, purified and used for subsequent digestion with EcoRI and BamHI restriction enzymes and insertion into pEGFP-N1 as described [27]. The resulting plasmid is schematically illustrated in Figure 1A. Competent *E.coli* JM109 cells were transformed with re-ligated vectors in order to amplify the plasmids. Bacteria were grown using kanamycin as selection antibiotic, and resistant clones were checked for their plasmid content by colony-PCR using CathB 49-1068 forward and reverse primers. Plasmid-DNA was prepared from overnight cultures. Both plasmids were sent for sequencing to verify correct DNA sequences (see Figure 1B).

### Transfection of thyroid cells

Transfection of FRT, KTC-1, HTh7 and HTh74 cells with pEGFP-N1, pCathB-eGFP or pCathB-C29A-eGFP was carried out using jetPEIMan (Qbiogene, Heidelberg, Germany), a mannose-conjugated linear polyethylene imine, which is able to compact DNA into positively charged particles followed by binding of the jetPEI-Man-DNA complexes to cell surface mannose-specific receptors and internalization through endocytosis. FRT and human thyroid carcinoma cells were seeded in 6-well plates on cover slips and transfected at 50-60% confluence. Transfection was performed according to the manufacturers instructions and by mixing jetPEI-Man and plasmid DNA at N/P ratios of 5 based on N-residues of the transfection reagent (7.5 mM) as compared to anionic phosphate of the plasmid DNA (3 nM/μg). The transfected cells were cultured for 24 hours under normal cell culture conditions. On the following day, transfection medium was exchanged for normal medium. The transfected cells were either analyzed directly through live-cell imaging with a confocal laser scanning microscope as described above or, in case of pCathB-C29A-eGFP transfected FRT cells, they were subjected to antibiotic selection with G418 (Merck KGaA, Darmstadt, Germany).

## Results

### Localization of cathepsin B in human thyroid tissue

**Figure 2:**
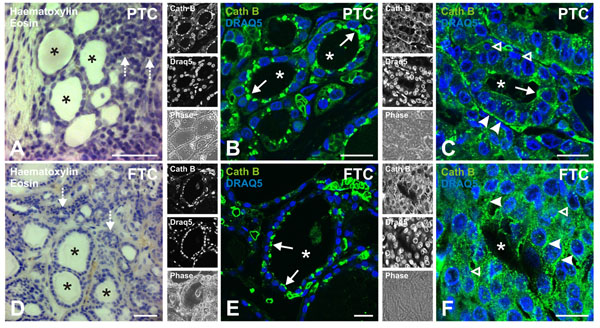
**Tissue architecture and distribution of cathepsin B in thyroid carcinoma *in situ***. Micrographs of haematoxylin and eosin stained tissue of thyroid tissue derived from patients with papillary (A) and follicular (D) thyroid carcinoma (PTC and FTC, respectively) was inspected by brightfield microscopy. PTC tissue displayed areas with intact follicle structures (A) as well as areas with a disorganized tissue structure (A, dashed arrows). In addition, PTC tissue displayed fibrovascular cores (A). FTC tissue also displayed areas with intact follicle structures as well as areas with a disorganized tissue structure (D, dashed arrows). Merged (B, C, E, F), single channel fluorescence and corresponding phase contrast micrographs (left panels) taken with the confocal laser scanning microscope of thyroid tissue from patients with PTC or FTC showing the endogenous distribution of cathepsin B (green, left panels, top). The nuclei were visualized through DRAQ5™ counter-staining (blue, left panels, middle). Note that cathepsin B was localized to vesicles close to the apical plasma membrane (arrows) in non-neoplastic areas of thyroid carcinoma tissue (B and E, respectively). Cathepsin B distribution was different in the disorganized, neoplastic areas of thyroid carcinoma tissue (C and F). The protease was immunolocalized to vesicles scattered in the cell periphery along the basal and lateral plasma membrane domains as well as scattered throughout the cytosol (C and F, arrowheads). Nuclei of cells in neoplastic areas displayed an abnormal morphology (C and F, open arrowheads). Follicle lumina are denoted by asterisks. Scale bars represent 50 µm in A and D, and 20 µm in B, C, E, and F.

Human tissue obtained from patients affected by follicular thyroid carcinoma (FTC) or papillary thyroid carcinoma (PTC) was analyzed in order to determine the general tissue architecture and the localization of endogenous cathepsin B. Haematoxylin and eosin-stained PTC tissue displayed normal thyroid histomorphology with variably sized follicles and colloid-containing lumina that were enclosed by a monolayer of epithelial cells (Figure 2A, follicle lumina denoted with asterisks). In addition, papillary stalks and disorganized neoplastic areas lacking clear follicular structures were abundantly detected in PTC-derived tissue (Figure 2A, dashed arrows). Heterogenous histomorphology was also detectable in haematoxylin and eosin-stained FTC-derived tissue that demonstrated both intact follicles (Figure 2D, follicle lumina denoted with asterisks), microfollicles and disorganized neoplastic areas (Figure 2D, dashed arrows). Cathepsin B was shown by immunostaining to be localized within vesicular structures located close to the apical plasma membrane in the non-altered areas of thyroid carcinoma tissue (Figure 2B and E, arrows). In the areas with altered morphology of the same tissue, however, cathepsin B-positive vesicles were detected only occasionally at the plasma membrane and appeared distributed throughout the cytosol of thyroid follicle cells (Figure 2C and F, arrowheads) that were further characterized by abnormal nuclear morphology and by less polarized states than thyroid cells in normal tissue areas (Figure 2C and F, open arrowheads). In addition, cathepsin B-containing vesicles were localized to the basolateral poles as highlighted by immunostaining of the basal plasma membrane domains of neoplastic cells in PTC (Figure 2C, arrowheads) and of the lateral plasma membrane domains in FTC tissue (Figure 2F, arrowheads), indicating non-directed cathepsin B secretion into the extrafollicular space.

This change in cathepsin B distribution from a prominent apical localization in normal to a basolateral localization in neoplastic areas is therefore not a feature of PTC alone [20], but also observed in FTC. This notion made us to hypothesize that protease trafficking is dramatically altered in thyroid carcinoma, which led us to analyze cathepsin B transport pathways in more detail in different thyroid carcinoma cell lines that are known to exhibit at least some features of differentiated thyrocytes although being transformed and representative of papillary and anaplastic thyroid carcinoma cells. Trafficking of cathepsin B in normal thyroid epithelial cells that are fully differentiated and exhibit a polarized phenotype, i.e. FRT cells, was studied for comparison.

### KTC-1, HTh7 and HTh74 as model cell lines to study cathepsin B trafficking in thyroid carcinoma

Thyroid carcinoma cells of variable aggressiveness differ in the degree of differentiation and polarization [30,31]. In this study, we have used the papillary thyroid carcinoma cell line KTC-1 as well as the anaplastic thyroid carcinoma cell lines HTh7 and HTh74 [22,23].

**Figure 3:**
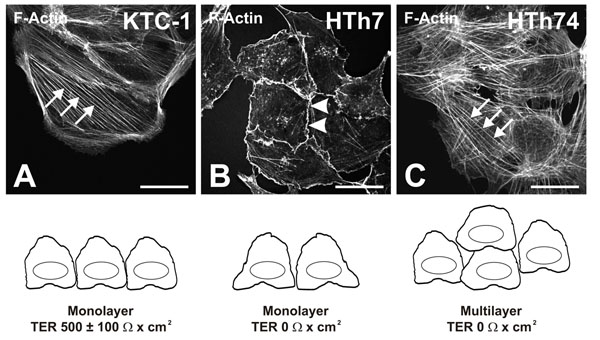
**Thyroid carcinoma cell lines as model systems**. Fluorescence micrographs of the papillary thyroid carcinoma cell line KTC-1 (A) and the anaplastic thyroid carcinoma cell lines HTh7 and HTh74 (B and C, respectively) taken with the confocal laser scanning microscope after fixation and phalloidin staining of the F-actin cytoskeleton. KTC-1 and HTh74 cells displayed stress fibers throughout the cytosol (A and C, arrows) as well as cortical F-actin underneath the plasma membranes. In HTh7 cells stress fibers were largely lacking (B) but cortical F-actin was displayed (B, arrowheads). KTC-1 cells grew in a monolayer fashion and established only a weak barrier as estimated by determination of the trans-epithelial electrical resistance (TER), whereas HTh7 cells formed non-tight monolayers and HTh74 cells grew in multilayers. Scale bars represent 20 µm.

KTC-1 and HTh74 cells displayed prominent stress fibers running throughout the cytosol when F-actin was stained with FITC-phalloidin (Figure 3A and C, arrows), whereas HTh7 cells have lost the ability to tightly adhere to the substratum and lacked actin stress fibers (Figure 3B). Thus, KTC-1 and HTh74 cells in particular were polarized and should therefore, in principle, have the ability to establish and maintain an epithelial monolayer, which was analyzed by determination of the trans-epithelial electrical resistance (TER) as a measure of monolayer tightness [32]. KTC-1 cells indeed formed a confluent monolayer but displayed a low TER of 500 ± 100 Ω x cm^2^. In contrast, HTh7 and HTh74 cells were able to hyper-proliferate in culture, thereby forming mono- and multi-layers which were not tight since no TER was measurable. Therefore, KTC-1 cells must be considered to maintain contact-inhibition to some extent, which was clearly lost in HTh74 cells despite their ability to adhere to the substratum in a polarized fashion. In addition, adherens and tight junction proteins such as E-cadherin, claudin-1 and occludin have been detected at the lateral plasma membrane domains of KTC-1 cells (our own unpublished observations; KBr, Laura Panavaite, ST, all Bremen), thus further supporting maintenance of epithelial polarity to some extent in this cell line.

Because of the morphological and functional appearance of KTC-1 cells as well as due to the lack of contact-inhibition in hyper-proliferative HTh7 and HTh74 cells, we considered these cell lines suitable to represent distinct stages in epithelial-to-mesenchymal transition with HTh7 cells being the most progressed toward the mesenchymal phenotype, but HTh74 cells being the most transformed with respect to proliferation and loss of contact inhibition.

### Cathepsin B is secreted from KTC-1 cells

We have previously shown that cathepsin B is one of the main if not the major cysteine peptidase active in KTC-1, HTh7 or HTh74 cells, and that its predominant expression pattern is vesicular in these thyroid carcinoma cell lines [21]. In this study, we were interested in the investigation of the 3-dimensional distribution of cathepsin B-containing vesicles through optical sectioning by means of confocal laser scanning microscopy in order to approach determination of the transport pathways of the endogenous protease before analyzing trafficking of GFP-tagged chimeras of active and inactive cathepsin B.

**Figure 4:**
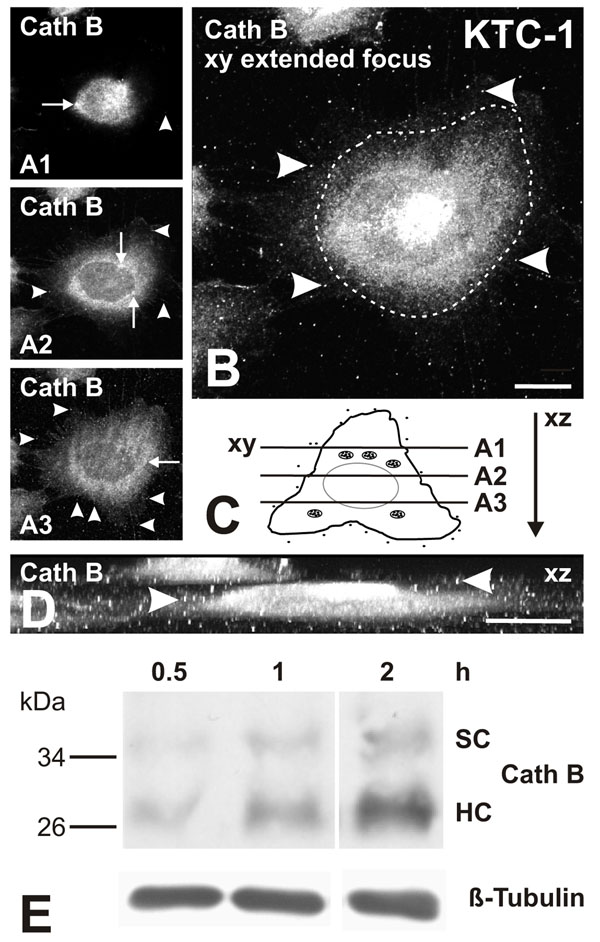
**Secretion of endogenous cathepsin B from KTC-1 cells**. Single channel fluorescence micrographs of cathepsin B localization in KTC-1 cells as detected by immunolabelling and confocal laser scanning microscopy (A, B, D). Three-dimensional distribution of cathepsin B representative signals is sketched in C. Single optical sections taken in xy were obtained in different focal planes (A1-A3, cf. lines in C) and compiled in a zero-projection as an extended focus (B) as well as in xz-direction as a side view (D). Cathepsin B displayed a reticular and vesicular staining pattern throughout the cell, and it appeared extracellularly in a dotted fashion due to its re-association with the plasma membrane (A-D, arrowheads). Note that cathepsin B secretion was non-directional. The dashed line in B denotes the cell circumference. Scale bars represent 10 µm. (E) TCA-precipitated and SDS-PAGE separated proteins from conditioned media of KTC-1 cells were immunoblotted with anti-cathepsin B antibodies. Note the presence of mature forms of cathepsin B, i.e. single chain (SC) and heavy chain (HC) cathepsin B in conditioned media and their increasing amounts with time. Immunoblotting of beta-tubulin in the corresponding whole cell lysates were used as loading controls.

Optical sections (xy) were taken at different focal planes of cultured KTC-1 cells in z-direction, i.e. perpendicular to the cellular poles attaching to the substratum. Cathepsin B was detected by immunofluorescence staining within reticular structures and vesicles gathering in the peri-nuclear region (Figure 4A1-A3, arrows) and in a dotted pattern (Figure 4A1-A3, B, D, arrowheads). When the sections of an entire z-stack were compiled and reconstructed in xz, i.e. displayed as side view as schematically indicated in 4C, it became evident that cathepsin B-positive structures were localized in a dotted fashion in close proximity to the cell periphery (Figure 4B and D, arrowheads). This close proximity to the cell surfaces is most probably an indication of cathepsin B being released either towards the apical pole (representative of into the follicle lumen) or towards the basal side (representative of towards the extrafollicular space) and its subsequent re-association with the plasma membrane of KTC-1 cells.

Therefore, we next analyzed the media conditioned by KTC-1 cell cultures for possible occurrence of secreted forms of cathepsin B. In fact, KTC-1 cells were able to secrete mature, proteolytically active cathepsin B, because both, the single chain (SC) and the heavy chain (HC) of the two-chain form of cathepsin B were detectable in the conditioned media (Figure 4E). The levels of both, single and heavy chain cathepsin B increased steadily over time, indicating constant secretion of mature forms of cathepsin B from KTC-1 cells.

### Visualization of active cysteine cathepsins in HTh74 cells

**Figure 5:**
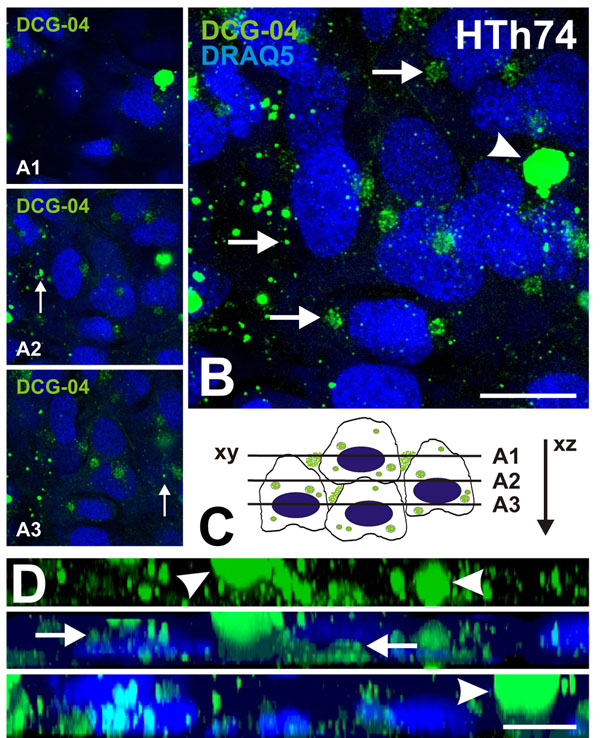
**Visualization of active cysteine cathepsins in HTh74 cell cultures**. Merged fluorescence micrographs of HTh74 cells growing in multilayers as detected after incubation with the fluorophore-conjugated activity based probe DCG-04 (green signals), counter-staining of nuclear DNA with DRAQ5™ (blue signals), and confocal laser scanning microscopy (A, B, D). The activity based probe DCG-04 binds to the active site of mature cysteine peptidases in a 1:1 ratio and visualizes proteolytically active cysteine proteases, only. Three-dimensional distribution of cysteine peptidase representative signals is sketched in C. Single optical sections taken in xy were obtained in different focal planes (A1-A3, cf. lines in C) and compiled in a zero-projection as an extended focus (B) as well as in xz-direction as a side view (D). Active cysteine peptidases were localized to variably sized vesicles throughout the cell (arrows). In addition, active cysteine peptidases were secreted from HTh74 cells and associated in aggregates with the plasma membrane (arrowheads). Note that active cysteine peptidases were secreted in a non-directional fashion at all poles of HTh74 thyroid carcinoma cells (D, arrowheads). Scale bars represent 10 µm.

In order to visualize active cysteine cathepsins in thyroid carcinoma cell lines, we used the activity based probe DCG-04 that binds covalently to active cysteine peptidases in a 1:1 ratio [26]. In HTh74 cells, proteolytically active cysteine peptidases were shown to be distributed throughout the cells in vesicles of various sizes (Figure 5A1-A3 and B, arrows). In addition, active cysteine peptidases were labeled with DCG-04 in differently sized aggregates that were localized in the extracellular space at both poles in between cells throughout the multi-layered cultures (Figure 5D, arrowheads). In particular, large DCG-04 positive cysteine peptidase-containing aggregates were found abundantly in association with the surfaces of HTh74 cells of the different layers (Figure 5B and D, arrowheads, schematically depicted in C). It was taken special care to wash the DCG-04 treated cells thoroughly before microscopy to avoid that aggregates would increase in size artificially during mounting for live-cell microscopy. Because the aggregates were visible in all focal planes above, in between, and below the multi-layers of HTh74 cultures (Figure 5D, green signals) and since they were not co-stained with DRAQ5™, we considered these structures as resulting from secretion of active cysteine peptidases rather than being derived from dead cells.

### Construction of a cathepsin B-C29A-eGFP coding vector

For *in vivo* analyses of cathepsin B-trafficking, vectors coding for different eGFP chimeras were used. As a control for normal trafficking of cathepsin B, the cDNA for cathepsin B from FRTL-5 cells, was cloned into the pEGFP-N1 vector [27]. To test whether the proteolytic activity of cathepsin B would affect its transport to distinct compartments, i.e. whether intrinsic sorting signals of the active enzyme would be a prerequisite of proper trafficking, the cDNA sequence coding for rat cathepsin B was altered by site-directed mutagenesis. The modified cDNA was cloned into pEGFP-N1, thereby constructing a vector coding for an inactive cathepsin B-C29A-eGFP chimera (Figure 1A).

The sequences of the inserted DNA for pCathB-eGFP and pCathB-C29A-eGFP revealed identical nucleotide sequences coding for cathepsin B, except the specifically inserted mutations, i.e. the codon exchange at position 421-423 for the cysteine to alanine exchange (Figure 1B, sequence 1) as well as a restriction site omission at position 406-408. The so-called C-terminal extension of cathepsin B, normally at amino acid positions 334-339, which is not needed for protease function [33] was lacking in both constructs, because both were linked to the eGFP portion by a 6-amino-acid spacer peptide instead.

### Expression of eGFP-tagged active and inactive cathepsin B in rat thyroid cells

**Figure 6:**
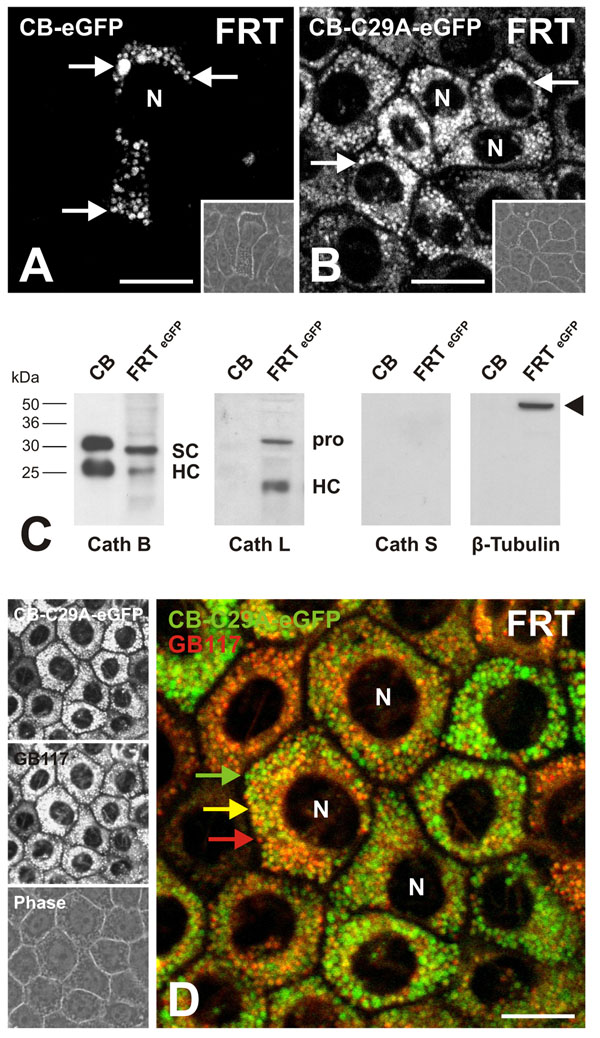
**Expression of cathepsin B-eGFP and cathepsin B-C29A-eGFP chimeras in normal thyroid epithelial cells.** Single channel fluorescence (A and B, left panels in D), merged (D) and corresponding phase contrast micrographs (insets in A and B, left panel, bottom, in D) taken with the confocal laser scanning microscope of FRT cells after transfection with plasmids coding for eGFP-tagged active (A) and inactive cathepsin B (B and D) as indicated. The expression pattern of both chimeric proteins was vesicular in FRT cells (arrows in A and B, green signals in D). The endogenous levels of mature cathepsins B, L and S (SC – single chain, HC – heavy chain) were estimated through immunoblotting and densitometry analysis and the cathepsins B:L:S ratio was shown to be 7:3:0 (C). The cathepsin levels were normalized against β-tubulin (C, arrowhead) that was used as a control for equal loading. The quenched activity based probe GB117 became fluorescent upon reaction with active cysteine peptidases and exhibited a vesicular staining indicative of active cysteine cathepsins in endo-lysosomal compartments (red signals in D). Note that the inactive cathepsin B-C29A-eGFP protein was localized within the same (yellow signals) or different vesicles (green signals) of normal rat thyroid epithelial cells (D), ruling out the existence of sorting signals intrinsic in cathepsin B’s primary structure. N denotes nuclei. Scale bars represent 20 µm.

FRT cells, a thyroid epithelial cell line derived from Fisher rats, form monolayers with tight and adhesive junctions separating apical and basolateral plasma membrane domains from each other. Therefore, they are an excellent model system to study the morphological properties of thyrocytes, which are highly polarized *in situ*[27,34]. FRT cells were transfected with pCathB-eGFP and pCathB-C29A-eGFP by means of jetPEI-Man. The expression of cathepsin B-eGFP as well as of the active-site mutant cathepsin B-C29A-eGFP showed prominent signals of the chimeric proteins within numerous vesicles that were reminiscent of endo-lysosomal compartments because of their accumulation within the peri-nuclear regions of FRT cells (Figure 6A and B, arrows). Importantly, the cathepsin B-eGFP chimeric protein has been shown not to be over-expressed in these cells [27]. Hence, in normal rat thyroid epithelial cells, eGFP-tagged cathepsin B and its active-site mutant were destined to endo-lysosomes, thereby ruling out that the active site mutation affected the structure and folding of cathepsin B in such a way that it would induce major alterations in its cellular transport pathways.

However, by eGFP-tagging alone it could not be determined whether identical vesicles contained both, the active protease cathepsin B (or any other active cysteine cathepsin) and its inactive counterpart cathepsin B-C29A-eGFP. Therefore and in order to directly visualize active cysteine cathepsins versus inactive cathepsin B, pCathB-C29A-eGFP-transfected FRT cells were additionally labeled with activity based probes as reporters of proteolytic activity of cysteine peptidases. In this case we used GB117, a quenched Activity Based Probe (qABP) that reacts primarily with the active forms of cysteine cathepsins B, L, and S [25]. The big advantage of using a qABP is that it contains a fluorescence donor- and a quencher group keeping it non-fluorescent before binding to and reacting with an active cysteine peptidase [6,25]. Upon covalent attachment of the qABP by reacting with the active-site residues of an active protease molecule, the quencher is released and fluorescence is exhibited. Hence, GB117 provides a tool to analyze whether inactive cathepsin B-C29A-eGFP is transported to vesicles that also contain active proteases, or, instead, whether the active site mutant counter-part of cathepsin B displayed sorting signals that would enable transport to a different vesicle population which would not contain any active cysteine proteases.

However, since GB117 has been shown to have a relative selectivity for cathepsin L over cathepsins B and S [25], we analyzed the amounts of these three cathepsins in whole cell fractions of FRT cells transfected with pEGFP-N1 vectors, i.e. lacking the cDNA coding for active or inactive cathepsin B, by immunoblotting. The amounts of mature cathepsin B (single chain plus heavy chain normalized to ß-tubulin) exceeded those of cathepsin L by more than 2-fold while cathepsin S was not detectable at all (Figure 6C). We can therefore conclude that GB117 evoked signals in FRT cells would derive primarily from its interaction with cathepsin B.

Next, cathepsin B-C29A-eGFP expressing FRT cells were analyzed by live-cell imaging after incubation with GB117 for 3 hours at normal cell culture conditions. The labelling of active cysteine peptidases with GB117 in FRT cells expressing inactive cathepsin B-eGFP chimeras resulted in a vesicular staining pattern, indicative for the presence of active cysteine proteases in endo-lyososomal compartments (Figure 6D, red signals). Furthermore, the GB117-labeled molecules co-localized with cathepsin B-C29A-eGFP chimeras within these vesicular structures, as was obvious from the yellow signals resulting from overlapping of red and green signals (Figure 6D, yellow signals). However, some vesicles were positive for cathepsin B-C29A-eGFP but lacked the signal for GB117 (Figure 6D, green signals). Thus, eGFP-tagging of the inactive cathepsin B mutant form as well as qABP-tagging of active cysteine proteases resulted in the notion that most endosomes and lysosomes of normal, polarized thyroid epithelial cells contained mixtures of active and inactive cysteine proteases thereby ruling out that active and inactive cathepsin B were sorted into distinct vesicle populations. These results indicated that sorting signals are unlikely to exist in the vicinity of the active site cleft of cathepsin B.

### Localization of active and inactive cathepsin B fused to eGFP in thyroid carcinoma cell lines

**Figure 7:**
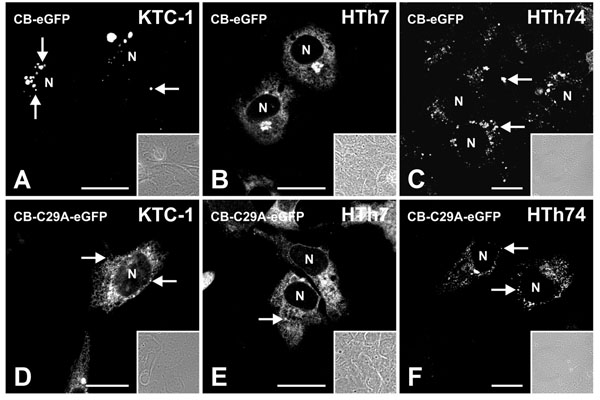
**Expression of cathepsin B-eGFP and cathepsin B-C29A-eGFP chimeras in thyroid carcinoma cells.** Single channel fluorescence (A-F) and corresponding phase contrast micrographs (insets) taken with the confocal laser scanning microscope of KTC-1 (A and D), HTh7 (B and E) and HTh74 cells (C and F) after transfection with plasmids coding for eGFP-tagged active (A-C) and inactive cathepsin B (D-F) as indicated. The localization of the eGFP-tagged cathepsin B was vesicular in KTC-1 and HTh74 cells (A and C, arrows), whereas it was retained in the Golgi of HTh7 cells (B). In contrast, the eGFP-tagged active site mutant counter-part of cathepin B was localized within reticular structures, the Golgi apparatus and a few vesicles (arrows) of KTC-1 (D) and HTh7 cells (E), while it was mainly localized to vesicles of HTh74 cells (F), indicating that the inactive mutant form of cathepsin B is transport-competent in principle. N denotes nuclei. Scale bars represent 10 µm.

Next, we were interested to determine whether cathepsin B trafficking is altered in thyroid carcinoma cells in comparison to normal thyroid epithelial cells. The active cathepsin B-eGFP chimeric protein was localized to vesicular structures in KTC-1 and HTh74 cells (Figure 7A and C), i.e. in a similar distribution pattern as that observed for endogenous cathepsin B. Thus, cathepsin B-eGFP is likely following the same transport pathway as endogenous cathepsin B. HTh7 cells, on the other hand, displayed an accumulation of CB-eGFP in the endoplasmic reticulum (ER) and was retained even more prominent in the Golgi apparatus (Figure 7B). This localization pointed to a transport defect of this thyroid carcinoma cell line, because the cathepsin B pattern observed by expression of the GFP-tagged protease was reminiscent to the predominant reticular staining pattern with only few vesicular structures that were immunolabeled in KTC-1 cells with cathepsin B-specific antibodies (see, Figure 4).

When the inactive cathepsin B-C29A-eGFP chimeras were expressed in KTC-1 and HTh7 cells, the green fluorescence was abundant in the endoplasmic reticulum (Figure 7D and E, arrowheads) but mostly absent from endo-lysosomes. In contrast, HTh74 cells expressing the inactive cathepsin B-C29A-eGFP chimeric protein still displayed a vesicular staining pattern resembling the peri-nuclear pattern of endo-lysosomal compartments (Figure 7F).

## Discussion

Cathepsin B processes thyroglobulin under physiological conditions in the extracellular follicle lumen as well as in endo-lysosomal compartments which is followed by the release of thyroid hormones from the thyroid gland [15-17]. Hence, transport of cathepsin B to the apical plasma membrane domain of normal thyroid epithelial cells is a prerequisite for its TSH-stimulated secretion into the follicle lumen in order to maintain thyroid homeostasis [19]. In pathological conditions, however, such as papillary thyroid carcinoma, cathepsin B has been localized to the basement membrane [20]. Here we provide evidence that such re-routing of cathepsin B transport from apical-to-basolateral poles is a hallmark also of neoplastic cells in FTC (see Figure 2). Therefore, we propose that cathepsin B transport towards basal poles is characteristic for cells in both, papillary and follicular thyroid carcinoma, whereas an apical-directed transport that is characteristic for thyrocytes of normal thyroid tissue, is also still displayed in cells of PTC and FTC-derived tissue areas with intact follicle structures. This notion motivated us to analyze the pathways resulting in altered cathepsin B trafficking and leading to its secretion into the extrafollicular space, which most probably enhances the invasive potential of thyroid carcinoma cells due to cathepsin B's ability to degrade ECM components [1,10-12,35,36].

The data achieved by 3-dimensional immunolocalization of endogenous cathepsin B and experiments employing activity based probes indicated that the thyroid carcinoma cell lines investigated in this study were characterized by cathepsin B trafficking that is destined to endo-lysosomes and, in addition, that cathepsin B is secreted into the extracellular space in a proteolytically active form (see Figures 4 and 5). Moreover, secretion of cathepsin B and related cysteine peptidases from KTC-1 and HTh74 cells was non-directed. We conclude that active cysteine peptidases are likely to reach extrafollicular locations in thyroid carcinoma tissue.

From the trafficking studies with GFP-tagged chimeras, it can be deduced that the active site mutant of cathepsin B, which is transport competent and reaches endo-lysosomes of FRT and HTh74 cells, is retained in the endoplasmic reticulum of KTC-1 and HTh7 cells (see Figures 6 and 7). Furthermore, eGFP-tagged wild type cathepsin B was retained in the Golgi of HTh7 cells. Hence, trafficking of cathepsin B is largely independent of signals intrinsic in the primary structure of the protease, rather transport pathways differ in the thyroid cell lines tested with trafficking defects being more prominent in the thyroid carcinoma cell lines KTC-1 and HTh7, while HTh74 cells remained transport competent and sorted cathepsin B into endo-lysosomes. This is likely to be the prerequisite for the massive secretion of cysteine peptidases like cathepsin B into the extracellular space of HTh74 cell cultures (see Figure 5) and it is likely to explain, why this cell line in particular has lost its contact inhibition and acquired an invasive phenotype.

### Protease transport in mammalian cells

The molecular mechanisms underlying protease trafficking to their points-of-action have been studied in a variety of tissues and cell types [6,27,37-39]. However, the transport of proteases in mammalian cells is still not fully understood up until today [1]. Cysteine cathepsins, i.e. endo-lysosomal proteases that may also act extracellularly, are synthesized as inactive pre-pro-enzymes at the rough ER (rER). There, the signal peptide is cleaved-off co-translationally. In the oxidizing milieu of the ER lumen, disulfide bridges are formed with the help of protein disulfide isomerase (PDI), an ER-resident enzyme, assisting in the correct folding of the proteins. Further, N-glycosylation of the synthesized proteases may be performed upon recognition of an Asn-X-Ser/Thr-Y motif (amino-acids given in three-letter code, with ‘X’ indicating any amino-acid, and ‘Y’ indicating any amino-acid except proline) by means of oligosaccharyl transferase. The pro-forms of cysteine cathepsins are further transported to the Golgi apparatus, where the zymogenes are modified in terms of N-linked oligosaccharide processing resulting in the addition of mannose-6-phosphate residues by a phosphotransferase and a phosphodiesterase. The mannose-6-phosphate tags are recognized in the *trans*-Golgi network (TGN) by highly specific mannose-6-phosphate receptors (M6P-R), which sort the pro-forms of cysteine proteases directly to the endo-lysosomal compartments [40]. However, some M6P-tagged proteins like for instance thyroglobulin or the aspartic lysosomal protease cathepsin D, escape endo-lysosomal targeting in thyroid epithelial cells and become secreted instead [41,42].

These results are similar to observations made in lysosomal storage diseases, such as I-cell disease, where it was shown that transport pathways of lysosomal enzymes may differ tremendously with respect to the cell type. For instance, I-cell disease patients lack an enzyme responsible for the addition of the M6P-tag, i.e. phosphotransferase, thus the lysosomal enzymes are not transported to the endo-lysosomal compartments, but become secreted [39]. The mis-routing of lysosomal enzymes was also examined in fibroblasts isolated from mice deficient in M6P-receptors and displaying an I-cell disease-like phenotype [43]. Interestingly, isolated hepatocytes from the same mice exhibited the complete set of enzymes within their endo-lysosomal compartments [44] highlighting that alternative pathways of endo-lysosomal targeting exist. Furthermore, it has been shown that cathepsin B can reach peripherally located vesicles in cancer cells by a pathway that is independent of M6P and most probably driven by sorting signals located within the pro-peptide region of the enzyme [45].

Hence, even though compelling evidence for alternative trafficking mechanisms has been published, the underlying sorting signals or alternative transport routes were not fully elucidated until today (for review see [46]). An excellent model system for the study of transport differences are cells which are characterized by distinct plasma membrane domains thus polarized into a basolateral and an apical plasma membrane domain. In order to elucidate the mechanisms that trigger apically or basolaterally-directed transport, Madin-Darby canine kidney cells (MDCK) or the thyroid epithelial cell line FRT have been intensively studied. Interestingly, FRT cells transport plasma membrane proteins to opposite cell poles as MDCK cells, even though both cell lines are polarized and display apparently morphological features of differentiated epithelial cells [27,34,47,48]. Interestingly, the precise mechanisms that explain why e.g. transmembrane proteins are inserted into either the basolateral or the apical plasma membrane domain of MDCK or FRT cells, respectively, remain elusive. However, because thyrocytes are able to perform vesicular protein transport to opposite cell poles, they qualify as excellent models in order to study protein trafficking in epithelial cells.

### GFP-tagging and activity based probes as tools to study protease trafficking in thyroid epithelial and carcinoma cells

Previously, we have constructed a mammalian expression vector encoding cathepsin B-eGFP that proved suitable for trafficking studies of cathepsin B in the fully differentiated and polarized rat thyroid epithelial cell line FRT as well as in TSH-responsive FRTL-5 cells [27]. This vector can also be used to analyze cathepsin B transport in Chinese Hamster Ovary cells and in a number of other cell types indicating that eGFP tagging of cathepsin B does not grossly alter its trafficking in mammalian cells. More recently, our original pCathB-eGFP vector has been modified in the eGFP portion in order to improve signal-to-noise ratios [49] and it was sub-cloned into a modified plasmid for tissue-specific expression under the control of the A33-antigen promoter [50]. In these cases, the cathepsin B-encoding sequence of the original vector was not altered.

In contrast, here we describe the construction of a vector coding for an inactive mutant counter-part of cathepsin B, in which the active site cysteine was substituted for an alanine. It was taken care to exchange cysteine with alanine instead of the more likely exchange of cysteine with serine (sulfhydryl side chain would then be exchanged by hydroxyl group), because we wanted to exclude the possibility of creating a serine protease-like protein by site directed mutagenesis of the cDNA coding for the cysteine peptidase cathepsin B. A serine exchange could have meant to create a catalytic dyad consisting of serine and histidine. Hence, our site-directed mutagenesis and cloning strategy aimed at the generation of an inactive enzyme with subtle changes in the active site cleft. The goal was to modify the primary structure of cathepsin B in such a way that the protein would still fold properly and thus, would not induce an unfolded protein response due to mis-folding and retention in the ER. In fact, these aims were achieved as is obvious from the observation that cathepsin B-C29A-eGFP chimeras proved fully transport-competent in the normal thyroid epithelial cell line FRT (see Figure 6), where it reached endo-lysosomes. In addition, the active site mutant counterpart of cathepsin B was sorted into endocytic compartments of the thyroid carcinoma cell line HTh74 (see Figure 7).

The cathepsin B-eGFP and cathepsin B-C29A-eGFP chimeric proteins were not only expressed in normal thyrocytes and in thyroid carcinoma cells, rather cathepsin trafficking was also investigated in combination with the activity based probe GB117 [13,25] in order to specify its sorting into transport vesicles. Hence, several aspects of protease transport were addressed in this study. (i) We analyzed whether active cysteine proteases are directed to vesicles different from those that are reached by inactive proteases. Thus, mature enzymes would display specific sorting signals to direct them into distinct sub-cellular compartments. (ii) As an alternative explanation of re-routing of cathepsin B transport in thyroid carcinoma cells, it was tested whether cathepsin B can be transported differently when expressed in normal epithelial cells versus tumor-transformed cells. Thus, assuming that sorting of proteases is governed by the features of the different cell-types themselves.

We provide evidence for the notion that HTh74 cells, although representing anaplastic thyroid carcinoma cells, maintain transport competence and directed cathepsin B-eGFP in both versions, active and inactive, to endo-lysosomes. In this respect, HTh74 cells clearly resembled normal, non-transformed FRT cells that transported both chimeric proteins to identical destinations. However, the non-TSH receptor bearing anaplastic thyroid carcinoma cell line HTh7 and the papillary thyroid carcinoma cell line KTC-1 exhibited trafficking defects. Here inactive cathepsin B was retained within the ER and only the active cathepsin B-eGFP was transported further, i.e. up to the Golgi apparatus and to the endo-lysosomes, respectively.

## Conclusions

In this study, we showed that specific transport signals within the sequence of cathepsin B are unlikely to exist that would explain why thyroid carcinoma cells transport the cysteine protease differently than normal thyroid epithelial cells. Hence, future studies have to show differences between benign and malignant or highly invasive thyroid carcinoma cells. We propose that the differences reside in expression of e.g. distinct members of the Rab-protein family which are known to co-determine the directionality of protein transport in epithelial and carcinoma cells of non-thyroid origin [51-53].

In summary, we conclude that protease trafficking requires tight regulation in order to ensure proper physiological functions. In a pathological context, mis-routed proteases can cleave in a non-regulated manner, because they reach new locations and perform their actions under conditions different from what is considered ‘normal’. Finally, re-routing of proteases in cancer may well lead to altered proteolytic potencies in that proteases will encounter a variety of substrates which they would not have been able to interact with under physiological conditions. Hence, the action of proteases is decisive for normal and diseased functions of cells or tissues in many respects. In turn, the understanding of transport pathways of proteases in normal versus tumor cells still provides clues for elucidating drug targets in new therapeutic approaches.

## Competing interests

The authors declare that they have no competing interests.

## Authors' contributions

ST performed the *in situ* analysis, the cathepsin immunoblotting, the characterization and trafficking studies of the human carcinoma cells and contributed in drafting the manuscript. SJ performed the cloning and the trafficking studies of the rat thyroid cells and contributed in drafting the manuscript. HR performed the studies with activity based probes in carcinoma cells. GB and MB provided the activity based probes and gave advice on data interpretation. DF provided the human thyroid tissue and gave advice on data interpretation. KBr devised the study and its design and drafted the manuscript. All authors read and approved the manuscript.
